# Recovery of Bioactive Compounds from Pomegranate (*Punica granatum* L.) Peel Using Pressurized Liquid Extraction

**DOI:** 10.3390/foods10020203

**Published:** 2021-01-20

**Authors:** Paula García, Carolina Fredes, Inés Cea, Jesús Lozano-Sánchez, Francisco Javier Leyva-Jiménez, Paz Robert, Cristina Vergara, Paula Jimenez

**Affiliations:** 1 Departamento de Nutrición, Facultad de Medicina, Universidad de Chile, Santiago 8380453, Chile; paulajimenez@med.uchile.cl; 2 Departamento de Ciencias de la Salud, Carrera de Nutrición y Dietética, Facultad de Medicina, Pontificia Universidad Católica de Chile, Santiago 7820436, Chile; 3 Center for Systems Biotechnology, Fraunhofer Chile Research, Santiago 8580704, Chile; ines.cea@fraunhofer.cl; 4 Research and Development of Functional Food Centre, 18016 Granada, Spain; jesusls@ugr.es (J.L.-S.); jleyva@cidaf.es (F.J.L.-J.); 5 Department of Food Science and Nutrition, University of Granada, Campus Universitario s/n, 18071 Granada, Spain; 6 Departamento Ciencia de los Alimentos y Tecnología Química, Facultad de Ciencias Químicas y Farmacéuticas, Universidad de Chile, Santiago 8380492, Chile; proberts@uchile.cl; 7 INIA La Platina, Instituto de Investigaciones Agropecuarias, Santiago 8831314, Chile; cristina.vergara@inia.cl

**Keywords:** pomegranate by-products, pomegranate peel extract, polyphenols, punicalagin, antioxidant, RSM

## Abstract

Pressurized liquid extraction (PLE) is a clean and environmentally friendly alternative for the recovery of bioactive compounds from fruit by-products. Herein we focused on PLE for the extraction of bioactive compounds from pomegranate peel using a combination of pressurized water and ethanol. The main aim was to determine the optimal PLE conditions, i.e., ethanol percentage and process temperature, to obtain a pomegranate peel extract (PPE) with maximum total phenolic content (TPC), punicalagin content, and antimicrobial activity (AMA). The experimental design was conducted using a central composite design with axial points. Response surface methodology was applied to optimize the response variables using the desirability function. Multiple response optimization indicated a process temperature of 200 °C and ethanol of 77% as optimal conditions. The TPC and the punicalagin content of PPE-PLE obtained under optimal conditions were 164.3 ± 10.7 mg GAE/g DW and 17 ± 3.6 mg/g DW, respectively. Our findings support the efficacy of PLE on TPC recovery but not in punicalagin recovery. The AMA against *S. aureus* was 14 mm. The efficacy of PPE-PLE in food applications must continue to be studied in order to achieve adequate information on its potential for developing new food additives.

## 1. Introduction

The pomegranate (*Punica granatum* L., Lythraceae) tree is native to northern India and other areas bordering the Himalayas, but over the centuries its cultivation has spread throughout the Mediterranean basin and the Americas [1]. Pomegranate is a balausta fruit consisting of a hard pericarp and a spongy mesocarp containing juicy arils. Pomegranate is consumed as fresh arils or as processed products, e.g., fresh or concentrated juice, infusions, or jam [2]. The juice yield is less than half of the total weight of the fruit [3]. Like other fresh fruits, pomegranate has an inedible fraction that is discarded although which exact part is disposed of depends on the fruit cultivar and cultural preferences [4,5]. The discarded fraction is used to calculate the unavoidable fruit waste intensity [5]. Thus, both the low yield in juice extraction and the inedible fraction (i.e., pomegranate mesocarp and peel) may explain why pomegranate fruit produces high levels of loss and waste along the food supply chain worldwide. In the first stages, fruit processing creates large amounts of pomegranate by-products as waste, while in the last stage, consumption of fresh pomegranate arils in households also results in high levels of unavoidable waste.

Food loss and waste results in a misuse of resources, e.g., water, land, energy, fertilizer, while also giving rise to methane and CO_2_ emissions from the natural decomposition of food [6]. Moreover, food loss and waste generate economic and social impacts [7,8]. The 2030 agenda for the United Nations sustainable development goals (SDGs) set food waste reduction targets (SDG 12) [9]. Due to this, several countries have adopted strategies to move toward a circular economy, where the food supply chain must take care of its by-products and food waste [10]. Because fruit by-products are considered food waste, their economic value is low [2]. However, pomegranate by-products have added value as a source of bioactive compounds, demonstrating the potential for developing new food additives and reducing waste in the agri-food industry.

Previous studies have shown that pomegranate peel is an important source of phenolic bioactive compounds such as phenolic acids, flavonoids, and hydrolysable tannins (ellagitannins) [11,12,13,14,15]. Among ellagitannins, punicalagin is the major compound found in pomegranate peel [16]. Phenolic compounds found in pomegranate peel have been associated with a wide range of biological activities in in vitro and animal models [17,18,19]. Antimicrobial, antioxidant, antidiabetic, anti-inflammatory, anticarcinogenic, and cardiovascular protective activities have been associated with ellagitannins such as punicalagin, punicalin, ellagic acid, and gallagic acid [20,21]. The antimicrobial activity of different types of pomegranate peel extract (PPE) has been studied mainly in vitro on pathogenic bacteria including *L. monocytogenes*, *S. aureus*, *E. coli*, *Yersinia enterocolitica*, *B. cereus*, *B. subtilis*, *S. enteritidis*, *S. typhy* and *Pseudomonas fluorescens* [22]. 

The profile and content of the bioactive compounds in PPE depend on the fruit cultivar, the pretreatment, and the extraction (e.g., solvent and method) procedure [18]. As a consequence, different types of PPE can show different degrees of antimicrobial activity.

Solid−liquid extraction is the most common procedure used to obtain bioactive compounds from pomegranate peel [16,23,24,25,26]. Specifically, methanol has been shown to be the most effective at extracting bioactive compounds from pomegranate peel in comparison to other organic solvents [16]. However, methanol extraction cannot be used in food applications [23]. 

Pressurized liquid extraction (PLE) has emerged as a novel technique to obtain bioactive compounds using both water and/or organic solvents in combination with elevated temperature and pressure [27]. PLE achieves fast, efficient, and selective extraction with a wide range of compound polarities, offering less extraction time and solvent consumption than conventional solid−liquid extraction [28]. Scarce information on the extraction of bioactive compounds from pomegranate peel using PLE has been reported [16,27]. In a first study, Cam and Hisil [16] showed that total phenolic content (TPC) using water in PLE was three-fold higher than using water in solid−liquid extraction (i.e., at atmospheric pressure conditions). A following study demonstrated that a combination of ultrasound and PLE was a clean and environmentally friendly alternative for extracting phenolic compounds from pomegranate peel [27]. 

The efficacy of PLE on the extraction of bioactive compounds from pomegranate peel using a combination of pressurized water and ethanol has not been evaluated. This study was therefore designed to gain new insight into this topic. The aim was to determine the optimal conditions, i.e., ethanol percentage and process temperature, to obtain a PPE-PLE with a maximum TPC, punicalagin content, and antimicrobial activity (AMA) using a multiple response optimization. The profile of phenolic compounds by high performance liquid chromatography–diode array detector–electrospray ionization–time of flight–mass spectroscopy (HPLC-DAD-ESI-TOF/MS), the antioxidant capacity (AC) and the cytotoxicity of the PPE-PLE obtained under optimal conditions were also characterized. This research contributes to the development of novel uses of pomegranate peel using a more environmentally friendly technique in order to reduce food waste in the pomegranate industry. 

## 2. Materials and Methods

### 2.1. Pomegranate Material Recovery

Pomegranate fruits (cv. Wonderful) were collected at the ripening stage (April 2017) from a commercial farm located in Vallenar (28°34′ South Latitude; 70°45′ West Longitude) in the Atacama Region of Chile. Fruit samples were taken randomly from the upper, middle and lower canopy, stored at 4 °C and processed within 24 h of collection. Pomegranate peel from fresh fruits was manually separated and dried by convection in an air-drying tunnel (no brand, built with a Tetlak motor) with a horizontal air flow rate of 2 m/s and 50% recirculation at 60 °C for 16 h. The dried product was ground in a knife mill (Wiley Mill, Model−2, A.H. Thomas Co., Swedesboro, NJ, USA) and passed through a 20 mesh (840 microns) sieve. The resulting pomegranate peel powder was stored in darkness and kept at room temperature until extraction. The proximate composition of pomegranate peel powder was determined, including moisture, proteins, ash and fat according to the AOAC official procedures [29]. The content of total dietary fiber was determined by enzymatic gravimetric method [30]. TPC was spectrophotometrically quantified using a Folin−Ciocalteu phenol reagent assay [31], and punicalagin content was determined by HPLC [32]. In TPC and punicalagin analysis, a pomegranate peel powder sample was treated with ethanol:water (40:60 *v*/*v*) for 3 h using a solid−liquid extraction.

### 2.2. Chemical Reagents

For the extraction procedure, double-deionized water with conductivity lower than 18.2 MV was obtained with a Milli-Q system (Millipore, Bedford, MA, USA) and ethanol (purity ≥ 99.9%) was obtained from Panreac (Barcelona, Spain).

In order to characterize phenolic compounds and antioxidant capacity, all chemicals used were of analytical reagent grade. HCl (purity ≥ 37%), NaOH, NaCl, KCl, NaH_2_PO_4_, KH_2_PO_4_, sodium carbonate anhydrous, acetic acid glacial, sodium acetate trihydrate, iron(III) chloride hexahydrate, Folin−Ciocalteu’s phenol reagent, 6-hydroxy-2,5,7,8-tetramethylchroman-2-carboxylic acid (Trolox), were purchased from Merck (KGaA, Darmstadt, Germany). Gallic acid (purity ≥ 98%), 2,4,6-Tris(2-pyridyl)-s-triazine (TPTZ), 2,2′-Azobis(2-methylpropionamidine) dihydrochloride (AAPH), and Fluorescein sodium salt were purchased from Sigma-Aldrich (St. Louis, MO, USA). 

For HPLC characterization, water (LC-MS grade LiChrosolv^®^), methanol, and acetonitrile (Liquid chromatography LiChrosolv^®^) were purchased from Merck S.A (Santiago, Chile). Standard of punicalagin (purity ≥ 98%) was purchased from Sigma-Aldrich (St. Louis, MO, USA).

Finally, for cytotoxicity analysis a Vybrant MTT Cell Proliferation Assay Kit (Thermo Fisher Scientific, Leicestershire, UK) was utilized.

### 2.3. Extraction of Phenolic Compounds from PPE by PLE

Extraction of the phenolic compounds by PLE was carried out in 34-mL extraction cells, containing a mixture of pomegranate peel powder (3.75 g) and sand (11.25 g) using a pressurized liquid extractor (Dionex ASE 350, Accelerated Solvent Extractor (Thermo Fisher Scientific, Leicestershire, UK)) at a pressure of 1500 psi for 20 min. The resulting extract (PPE-PLE) was filtered (0.22 μm PTFE membrane filters, VWR International, Atlanta, GA, USA) and stored absent of light at −80 °C.

### 2.4. Experimental Design for PPE-PLE

The extractions from pomegranate peel powder using PLE were performed using a central composite design (CCD) with axial points, following general Equation (1)

(1) $$Y=b_{0}+\sum_{i=1}^{2}b_{i}X_{i}+\sum_{i=1}^{2}b_{ii}X_{i}{}^{2}+\sum_{i=1}^{1}\sum_{j=i+1}^{2}b_{ij}X_{i}X_{j}$$

where *Y* was the response; subscripts *i* and *j* ranged from 1 to the number of variables (*n* = 2); *b*_0_ was the intercept term; *b_i_* values were the linear coefficients; *b_ii_* values were the quadratic coefficients; *b_ij_* values were the interaction of the cross-product coefficient, and *X_i_* and *X_j_* were the levels of independent variables.

Twelve experiments were performed using the independent variables of ethanol (10–90%) in the water:ethanol mixture and process temperature (55–185 °C). The dependent variables were TPC, punicalagin content, and AMA. Response surface methodology (RSM) was applied to optimize the response variables using the desirability function (DF), where 1 represented the maximization of each variable [33]. All experiments were conducted randomly to avoid systematic bias. The linear, quadratic, and interaction effects of the independent variables on the response variables were considered at a confidence level of 95% (Statgraphics Centurion XV, Version 15.1.02, StatPoint, Inc., Warrenton, VA, USA).

### 2.5. Characterization of PPE-PLE Obtained under Optimal Conditions

#### 2.5.1. Determination of TPC

The TPC was spectrophotometrically quantified using a Folin−Ciocalteu phenol reagent assay [31]. The absorbance of samples was measured at 765 nm, and the results were expressed as milligrams of gallic acid equivalents per gram of pomegranate peel in dry weight (mg GAE/g DW), according to a calibration curve (100–800 mg GAE/L, *R*^2^: 0.9967). All analyses were performed in triplicate.

#### 2.5.2. Determination of Punicalagin Content

Punicalagin was detected and quantified via high performance liquid chromatography (HPLC) using a Merck Hitachi L-6200 pump, a Waters 996 photodiode-array detector (DAD), and a C18 column (5 μm, 4.6 i.d. × 250 mm, Symmetry, Waters, Ireland) employing the method described by Zhang et al. [32] with some modifications. Briefly, to prepare the mobile phase, Solvent A (0.4% aqueous phosphoric acid) and Solvent B (acetonitrile) eluted according to the following multistep gradient: 0 min (5% B); 10 min (15% B); 30 min (25% B); 35 min (5% B). A measure of 20 µL of the sample was injected and the flow rate was 1.0 mL/min at room temperature.

The monitored wavelength was 360 nm for the detection and quantification of total punicalagin (calculated by the sum of the peak areas of punicalagin A and B), according to a calibration curve (12–200 mg punicalagin/L extract, *R*^2^: 0.9942). The results were expressed as milligrams of punicalagin per gram of pomegranate peel in DW (mg/g DW). All analyses were performed in triplicate.

#### 2.5.3. Determination of the Antioxidant Capacity (AC)

The Ferric Reducing Antioxidant Power (FRAP) assay was determined according to Benzie and Strain [34] with modifications. Briefly, a portion of an aqueous 10 mM solution TPTZ (2,4,6-tris(2-pyridyl)-s-triazine) reagent in 40 mmol/L HCl was mixed with the same volume of 20 mmol L-1 FeCl_3_ · 6H_2_O and a 10-fold higher volume of acetate buffer, pH 3.6 (3.1 g sodium acetate and 16 mL acetic acid/L). The mixture was then incubated at 37 °C for 10 min. A portion (2700 μL) of the Fe^3+^-TPTZ mixture and 30 μL of each sample (or standard or water for blank) were combined and diluted to 270 μL with deionized water and then incubated at 37 °C for 30 min. Next, an aliquot of 225 µL of solution was transferred to a microplate (96 well, elisa plate) and the measured absorbance was 593 nm. Results were expressed as µmol of Trolox equivalents per g of pomegranate peel in DW (µmol TE/g DW), according to a calibration curve (200–1600 µM Trolox, *R*^2^: 0.9958). Each analysis was carried out in triplicate. 

The Oxygen Radical Absorbance Capacity (ORAC) assay was determined according to Dávalos et al. [35]. Results were expressed as µmol of Trolox equivalents per g of pomegranate peel in DW (µmol TE/g DW), according to a calibration curve (6.25–100 µM Trolox, *R*^2^: 0.9958). Each analysis was carried out in triplicate.

#### 2.5.4. Identification of Phenolic and Other Polar Compounds in PPE-PLE by HPLC-DAD-ESI-TOF/MS

Samples of PPE-PLE were analyzed in a high-performance resolution liquid chromatography (HPLC) system (Agilent Technologies, Waldbronn, Germany) equipped with a vacuum degasser, autosampler, binary pump, and diode-array-detector (DAD). This equipment was coupled to a time of flight mass spectrometer TOF (Bruker Daltonik, Bremen, Germany) equipped with an orthogonal electrospray (ESI) interface (model G1607 from Agilent Technologies, Palo Alto, CA, USA) operating in negative ion mode. The analytical column used was a C18 Zorbax Eclipse Plus (150 mm × 4.6 mm id, 1.8 µm, Agilent Technologies, Palo Alto, CA, USA).

The mobile phase was water acidified with 0.1% of formic acid (Solvent A) and methanol (Solvent B) eluted according to the following multistep gradient: 0 min (5% B); 42 min (95% B), 45 min (5% B) and 50 min (5% B). Then, 10 µL of the sample was injected and the flow rate was 0.4 mL/min at room temperature.

Polyphenols were detected by applying a mass range of 50–1500 *m*/*z*. The ESI source parameters were optimized and implemented as follows: capillary voltage of +4 kV; drying gas temperature, 210 °C; drying gas flow, 9 L min−1; and nebulizing gas pressure, 2.3 bar. The values of transfer parameters were: capillary exit, −120 V; skimmer 1, −40 V; hexapole 1, −23 V; RF hexapole, 80 V; and skimmer 2, −22.5 V. Moreover, to ensure proper calibration, the TOF mass spectrometer was externally calibrated using a 74900-00-05 Cole Palmer syringe pump (Vernon Hills, IL, USA) which was directly connected to the interface. The calibrant solution contained 10mM of sodium formate cluster. The mass data of the molecular ions acquired were managed using DataAnalysis 4.0 (Bruker Daltonics, Billerica, MA, USA) software, which uses a CHNO algorithm that increases the confidence in the suggested molecular formulas.

#### 2.5.5. Antimicrobial Activity (AMA)

The agar-well diffusion method was employed using Mueller-Hinton agar. The agar plate surfaces were inoculated by spreading 1 mL of the microbial inoculum over the entire agar surface. The inoculums were prepared using *Staphylococcus aureus* subsp. aureus (ATCC 25923) or *Escherichia coli* (ATCC 25922) and were suspended in sterile water and diluted to 10^6^ CFU/mL. Once the medium had solidified, wells with a diameter of 5 mm were punched aseptically with a sterile borer, and 70 µL of the PPE-PLE were placed into each well. Then, agar plates were incubated at 37 ± 1 °C for 24 h and AMA was evaluated by measuring the diameter of the inhibition zone of the tested bacteria. The results were expressed in mm.

#### 2.5.6. Cytotoxicity Assay

To determine the cytotoxic activity of PPE-PLE, an in vitro cell proliferation assay with an MTT (3-(4,5-dimethylthiazol-2-yl)-2,5-diphenyl tetrazolium bromide) kit was used on the human colorectal cancer cell line Caco-2 following the manufacturer’s instructions (Vybrant MTT). Cells were seeded onto a 96-well plate and incubated at 37 °C overnight with approximately 80% confluence, taking the cytotoxic compound SDS 0.2% as a positive control. Then, cells were incubated with 100 μL of PPE-PLE (in DW) at 3 different concentrations: 10, 50, 100 μg/mL, for 24 h. A portion of 10 µL MTT per well was added into the plate and incubated for an additional 3 h at 37 °C in the 5% CO_2_ incubator. The absorbance was measured in a microplate reader (Tecan Infinite^®^ 200PRO, Männedorf, Switzerland), at a wavelength of 570 nm. Cell viability was expressed as the percentage of viable cells compared to the control group without treatment. Experiments were performed in triplicate. 

#### 2.5.7. Statistical Analysis

The differences in cell viability using different PPE-PLE concentration was analyzed using a one-way ANOVA test for means comparison. When significant differences were found, the Tukey HSD (honest significant differences) multiple-comparison test (*p* ≤ 0.05) was applied. Analyses were performed with (Statgraphics Centurion XV, Version 15.1.02, StatPoint, Inc., Warrenton, VA, USA).

## 3. Results and Discussion

Pomegranate fruit processing is associated with unavoidable food waste, regardless of the manufacturers’ efficacy in managing and minimizing waste. Therefore, strategies to manage unavoidable food waste in the pomegranate industry are necessary to achieve SDGs and to move toward a circular economy [9,10]. Herein we focused on the treatment of a material (i.e., pomegranate peel) recycled from the pomegranate industry using PLE. Teigiserova et al. [36] proposed an updated hierarchy for food surplus and waste, where processing waste residues is equivalent to material recycling. Of note, material recycling actions appear in the center of the updated hierarchy [36]. Therefore, evaluating the efficacy of PLE on the recovery of bioactive compounds from pomegranate peel provides novel insights into alternative actions to mitigate food waste in the pomegranate industry. A previous study has reported that PLE is an environmentally friendly alternative for extracting phenolic compounds from pomegranate peel [27]. In this study, we showed new findings for the use of pomegranate peel powder as a recycled material, optimizing the PPE in order to obtain and characterize a PPE-PLE with maximum TPC, punicalagin content, and AMA.

### 3.1. Characterization of Pomegranate Peel Powder as Material Recycling

The unavoidable fruit waste intensity of pomegranate fruit was 57.4 ± 6.7%. The inedible fraction (i.e., pomegranate mesocarp plus pomegranate peel) can be used in material recycling. Specifically, pomegranate peel represented 10.5 ± 1.4% of the pomegranate-fruit fresh weight. After drying, the composition of the pomegranate peel powder was as follows: 3% moisture, 3.5% protein, 2.7% ash, 0.2% fat, and 60% available carbohydrates. Total dietary fiber was 30%, 14% soluble fiber and 16% insoluble fiber. Of note, the proximate composition of pomegranate peel was similar to that of pomegranate fruit, where the carbohydrate fraction predominated [37]. Nevertheless, pomegranate peel had a lower content of available carbohydrates than pomegranate fruit (i.e., 60% vs. 68%). In contrast, pomegranate peel contained higher dietary fiber content than pomegranate fruit (i.e., 30% vs. 18%). Indeed, following the daily recommended allowances (RDA) of dietary fiber in adults, pomegranate peel powder could therefore be considered a good source of dietary fiber [38]. TPC and punicalagin content were 125 mg GAE/g DW and 94 mg/g DW using solid−liquid extraction, respectively. These values were within the range described in the literature using solid−liquid extraction methods. 

### 3.2. Optimization of Extraction of Phenolic Compounds from Pomegranate Peel by PLE Using RSM

PLE is a sample preparation technique that combines elevated temperature and pressure with liquid solvents to achieve fast and efficient analyte extraction from a solid matrix [27,28]. Thus, temperature [X_1_] and solvent extraction (i.e., ethanol percentage [X_2_] in water:ethanol mixture) were selected as independent variables because both variables have demonstrated a significant effect on the extraction of phenolic compounds from pomegranate peel [16,18,27,28]. Pressure was not considered as an independent variable, so a high pressure of 1500 psi was constant in all of the experiments based on previous research on PLE [16,27]. In order to find the optimal conditions for obtaining a PPE-PLE, an RSM analysis was applied. Of note, the desirability function was comprised of the maximization of each variable and a multiple response optimization. Results of dependent variables in PLE from pomegranate peel and ANOVA results are detailed in Table 1 and Table 2.

**Figure 1:**
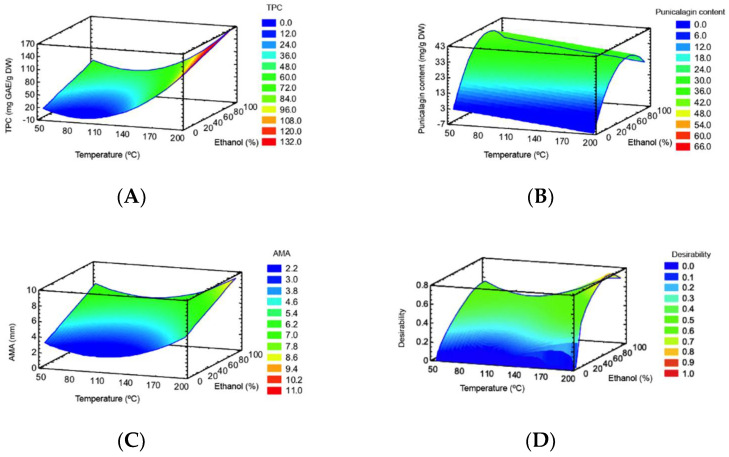
RSM plots for TPC (**A**), punicalagin (**B**), AMA (**C**) and multiple response optimization (**D**).

TPC ranged from 14.1 to 149.0 mg GAE/g DW (Table 1). The TPC was significantly affected by both lineal and quadratic forms (*p* < 0.05) of temperature, and the lineal form (*p* < 0.05) of ethanol (Table 2). The RSM plot (Figure 1A) showed that the highest TPC was obtained at the highest process temperature and intermediate ethanol percentage. This can be explained by the hydrolysis of polymeric compounds (e.g., ellagitannins) at high extraction temperatures resulting in monomeric phenolic compounds (e.g., ellagic acid) that were accounted in TPC measurement [39]. Moreover, ellagic acid glycosides that predominate in pomegranate peel are not affected much by temperature processing. For instance, thermal processing in red raspberry fruit did not greatly affect ellagic acid glycosides whereas free ellagic acid was more abundant after thermal processing [40]. The effect of ethanol percentage can be explained by the solvent polarity and the different polarity of bioactive compounds in the pomegranate peel matrix. This means that, as the ethanol percentage of the solvent increased, the extraction yield of polar compounds decreased [27]. Therefore, the recovery of compounds with a wide range of polarities present in pomegranate peels, ranging from polar (punicalagin and derivatives) to moderately polar (ellagic acid and derivatives) compounds may be accounted in TPC results. 

Punicalagin content ranged from 4.0 to 38.6 mg/g DW (Table 1). According ANOVA (Table 2), punicalagin content was significantly affected by both lineal and quadratic forms (*p* < 0.05) of ethanol. The lineal form (*p* < 0.05) of temperature also showed a significant effect on punicalagin content. The RSM plot (Figure 1B) shows that a high punicalagin content can be obtained at low process temperature at an intermediate ethanol percentage. 

The results showed that TPC and punicalagin content were different among the experiments. This behavior is characteristic of the PLE method, which presents differences in the selectivity of extraction [28].The extraction factors such as temperature and water:ethanol ratio have been associated with the dielectric constant. Indeed, the lower the dielectric constant (i.e., higher ethanol in the water-ethanol extracting solvent), the higher the flavonoids content [41].

The AMA ranged from 2.0 to 8.3 mm (Table 1) and was significantly affected by the quadratic form (*p* < 0.05) of temperature, and the lineal form (*p* < 0.05) of ethanol (Table 2). The RSM plot (Figure 1C) showed that the highest AMA was obtained at the highest process temperature and an intermediate ethanol percentage.

The coefficients of determination (*R*^2^) and *R*^2^-adjusted, and a nonsignificant lack-of-fit indicated that the mathematical models fitted well with the experimental data. Therefore, the models are suitable as a predictor of the TPC, punicalagin, and AMA. The multiple response optimization (Figure 1D) indicated a process temperature of 198.6 °C (i.e., the major axial point) and an intermediate ethanol of 76.6% as optimal conditions to obtain a PPE-PLE. In this study, RSM did not involve the maximum punicalagin content value of the experimental design, because the optimal conditions considered the optimization of several dependent variables (i.e., TPC, punicalagin and AMA) simultaneously.

### 3.3. Characterization of PPE-PLE Obtained under Optimal Conditions

#### 3.3.1. TPC and Punicalagin

The TPC and the punicalagin content of PPE-PLE obtained under optimal conditions was 164.3 ± 10.7 mg GAE/g DW and 17 ± 3.6 mg/g DW, respectively. Different values of TPC and punicalagin have been previously reported. Note, however, that the results among studies are not directly comparable, because of their different pomegranate genotypes, extraction and quantification methods. Based on solid−liquid extraction methods, Ambigaipalan et al. [42] reported a lower TPC (9.4 mg GAE/g of a pomegranate peel defatted sample) using acetone (70%). In addition, Fischer et al. [11] described a TPC of 101.9 mg GAE/g DW using aqueous methanol (80% *v*/*v*; 0.1% HCl) extraction. Contrarily, a higher TPC of 249.4 mg tannic acid equivalents/g DW using a mixture of methanol, ethanol, acetone and water, and a TPC of 432.7 mg GAE/g DW using ethanol:water (20:80 *v*/*v*) have been reported by Li et al. [43] and Rongai et al. [44], respectively. These studies showed the influence of extracting solvents on TPC, resulting in a wide range of TPC values. However, our results support the efficacy of PLE on the extraction of TPC from pomegranate peel using a combination of pressurized water and ethanol (76.6%).

Regarding punicalagin content, Rongai et al. [44] reported a much higher value (216.8 mg/g DW) using ethanol:water (20:80 *v*/*v*). Of note, another study has shown that a higher ethanol percentage in PLE extraction decreased solvent polarity, reducing the punicalagin extraction [27]. In our study, a combination of pressurized water and ethanol extended the use of PLE on the recovery of TPC from pomegranate peel under the experimental conditions, but did not enhance the recovery of punicalagin. 

#### 3.3.2. AC

The AC of PPE-PLE was 2265.6 ± 100.5 µmol TE/g DW measured by FRAP and 916.4 ± 102.0 µmol TE/g DW by ORAC. Polyphenol content in pomegranate peel extracts has been significantly correlated with AC, suggesting that the totality of polyphenols accounts for the AC observed [23,45]. Of note, the AC of PPE-PLE by FRAP was similar to the highest values obtained in pomegranate peel by Elfalleh et al. [24]. 

#### 3.3.3. Qualitative Characterization of Phenolic and Other Polar Compounds in PPE-PLE by HPLC-ESI-TOF-MS

**Figure 2:**
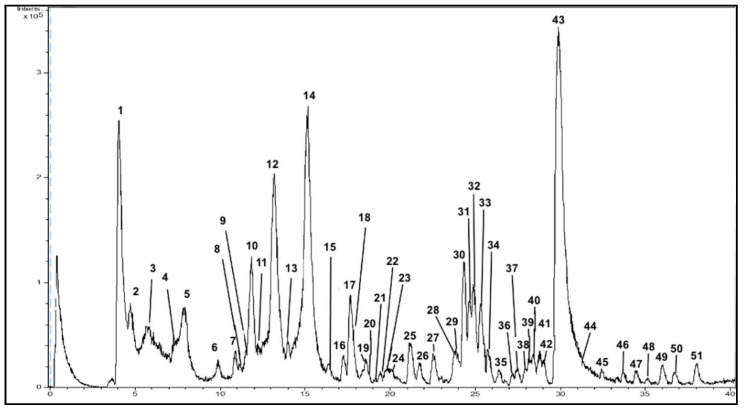
Representative HPLC-DAD-ESI-TOF/MS chromatogram of polar compounds in PPE-PLE.

The chromatogram for PPE-PLE obtained by the HPLC-ESI-TOF-MS method is shown in Figure 2. The identification was based upon an interpretation of their MS spectra provided by TOF-MS and the information suitable in the literature. Table 3 shows the tentative identification of each peak with their retention times (RT), experimental and calculated *m*/*z* and molecular formula. These compounds have been numbered according their elution order. The HPLC-ESI-TOF-MS method allowed the detection of 51 compounds. Among these, a total of 42 compounds were identified, and then classified into five groups based on their chemical structure. Unfortunately, nine minor compounds could not be identified with the method used. Further analysis using improved identification methodologies should be performed in order to identify these unknown compounds.

- Sugars

The method applied allowed for the identification of four sugars in PPE-PLE (compounds **1**, **2**, **11** and **13**). These compounds were eluted earlier in the chromatographic run due to their highly hydrophilic character. Some studies have attributed the sweetener property of fruits to glucose, fructose, and sucrose [46,47]. Furthermore, two sugar derivatives, compounds 11 and 13, were found at 12.8 and 14.2 min, respectively. These are related to hexose or pentose derivatives. Unfortunately, an accurate identification could not be achieved because while the MS equipment used was able to provide molecular formulas, it could not give functional group positions.

- Organic acids

According to the MS spectra provided by TOF-MS and the elution profile, three organic acids were identified: citric acid (compound **4**) and two derivatives. Compound **5** showed an elution time of 8.0 min enabling it to be identified as isocitric acid (*m*/*z* 191.0197) and compound **17** eluted at 17.7 min which provided a *m*/*z* at 219.0510. MS data characterized it as dimethyl ester citric acid [48].

- Phenolic acids

A total of eight phenolic acids were detected after examining the MS spectra, elution time, and available bibliography. Chlorogenic acid (compound **3**) with the molecular formula (C_16_H_18_O_9_) and a retention time of 5.9 min was identified. Compound **7** showed a *m*/*z* of 125.0244 and a deprotonated molecular formula C_6_H_5_O_3_ which identified it as phloroglucinol [49]. Compound number **10** was related to gallic acid, as previously described in other studies [11,50]. Additionally, another gallic acid derivative was found and associated to compound **30**. Specifically, this compound was related to gallic acid dimethyl ether since it gave the molecular formula C_9_H_10_O_5_. Compound **34** is a ferulic acid derivative (feruloyl sucrose), which was previously identified in other studies [11]. Furthermore, other phenolic acids corresponding to compounds **24, 35** and **42** were also detected. The first compound (**24**) was identified as *p*-coumaric acid hexoside, the first time this compound has been detected in pomegranate. Additionally, compound **35** presented a *m*/*z* of 293.1031 and a retention time of 26.4 min being identified as cinnamoyl rhamnoside. Compound **42** was also identified as homovanillic acid (C_9_H_10_O_4_). This compound was characterized on the basis that cinnamic acid and its derivatives were previously found in pomegranate peel [24,51,52].

Phenolic acids from pomegranate peel have shown the capacity to inhibit the activity of the angiotensin converting enzyme (ACE) that is implicated in blood pressure [21]. Gallic acid, has been associated with antidiabetic, anti-inflammatory, antioxidant and anticarcinogenic properties. Additionally, caffeic acid increases glucose uptake by rat adipocytes and mouse myoblasts [53].

- Flavonoids

Overall, six flavonoids were detected in PPE-PLE. The first eluted flavonoid (18.6 min) was catechin which was related to compound **19**. [54,55]. Compound **39** (28.1 min), which gave a deprotonated molecular formula of C_21_H_19_O_12_ enabled its identification as hyperoside. This flavonoid has previously been found in different pomegranate cultivars [50,56]. In addition, kaempferol (compound **47**) and two luteolin derivatives were detected and attributed to compounds **44** and **46**. They gave similar molecular formulas of C_21_H_19_O_11_ and C_20_H_17_O_10_, respectively. Therefore, it could be established that they were joined to a hexose and a pentose, characterizing compound **44** as luteolin hexoside and compound **46** as luteolin pentoside. Both luteolin hexoside and luteolin pentoside have previously been found in pomegranate [11]. Additionally, luteolin was tentatively identified as compound **49**, since it displayed a deprotonated molecular formula of C_15_H_9_O_6_ and has previously been described in the literature [50]. 

Flavonoids of pomegranate peel are associated with antioxidant properties and improving oral health, particularly in relation to gingivitis development [22,54].

- Hydrolysable tannins

The analytical data provided enabled 21 hydrolysable tannins to be identified in PPE-PLE, the most abundant chemical group found. Because of the broad variety of compounds belonging to this group, they were classified in two groups: gallotannins and ellagitannins. Regarding gallotannins, compounds **6** and **8** presented the same deprotonated molecular formula (C_13_H_15_O_10_) and a *m*/*z* ratio of 331.0671, characterizing them as galloyl-hexoside isomers. Isomer 1 and 2 were detected. In addition to these compounds, four additional tannins derived from gallic acid were also characterized. Thus, compound **18**, which displayed a molecular formula C_27_H_22_O_18_, was related to galloyl-HHDP-hexose [11]. Compound **20,** which presented a *m*/*z* of 483.0780, was tentatively identified as digalloyl hexoside. Additionally, another gallotannin was found with a retention time of 24.1 min. This compound was characterized as methyl gallate hexoside (compound **28**). Finally, the last gallotannin was compound **29,** related to gallagyldilactone [57]. 

Ellagitannins are predominant in pomegranate. Among them, some isomers of punicalagin were found. Indeed, compounds **9**, **12** and **14**, which showed the same molecular formula (C_24_H_14_O_15_), were related to punicalagin α, β and γ, respectively. Isomers α and β were identified earlier by following their elution order, as has been described in other studies [27,58]. However, isomer γ was identified for the first time in this study. In addition, compound **15** with a *m*/*z* of 469.0049 was assigned to valonic acid bilactone [11,51]. Compound **38** was associated with a valonic acid derivative, particularly, monodecarboxyvaloneic acid dilactone. Compound **25** gave a deprotonated molecule of *m*/*z* 633.0463 with the molecular formula C_27_H_21_O_18_ and eluted at 21.3 min. This peak was associated with corilagin, a compound previously described in pomegranate [59]. Likewise, compound **26** displayed a deprotonated molecule at a *m*/*z* of 291.0146 and a deprotonated molecular formula of C_13_H_7_O_8_. These results enabled its identification as brevifolin carboxylic acid which has previously been detected in pomegranate peel [11]. Furthermore, compound **33** displayed a molecular formula of C_12_H_8_O_6_ and it was characterized as brevifolin [60]. In addition, brevifolincarboxylic acid ethyl ester was found and assigned to compound **36** which gave a *m*/*z* of 319.0459. The occurrence of this compound in this extract may be caused by conditions applied during the PLE extraction procedure.

Finally, the MS spectra acquired characterized ellagic acid and five derivatives. The first eluted ellagic acid derivative was ellagic acid hexoside (compound **31**). Then, compound **32** (C_13_H_8_O_7_) was characterized as decarboxyellagic acid. Compounds **40** and **41** were identified as ellagic acid pentoside and elagic acid deoxyhexoside with the same elution order that has been previously reported in the literature [27]. Altogether, a deprotonated molecular formula of C_14_H_5_O_8_ was given by compound **43**. This compound, characterized as ellagic acid, comprised more than 50% of the total phenolic compounds in pomegranate peel, the highest intensity of all the compounds studied [61]. The last ellagitannin eluted in the chromatographic run was compound **48** which gave a *m*/*z* of 329.0303, identified as ellagic acid dimethyl ether. 

Ellagitannins, mainly punicalagin, are responsible for the antioxidant and antimicrobial activity of pomegranate peel extract. In addition, the anticarcinogenic effects and inhibition of inflammatory processes are attributed to punicalagin, ellagic acid and gallic acid [21].

#### 3.3.4. AMA

The AMA of PPE-PLE was evaluated on *S. aureus* and *E. coli* by measuring the diameter of the inhibition zone of the tested bacteria. AMA was only observed on *S. aureus* with an inhibition diameter of 14 mm. These results could be explained by the differences between Gram-negative and Gram-positive bacteria membrane. Gram-negative bacteria (e.g., *E. coli*) would be more resistant to polyphenolic extracts than Gram-positive bacteria (e.g., *S. aureus*), because Gram-negative bacteria possess a complex outer lipopolysaccharide membrane which slows down the passage of polyphenols [62]. The antimicrobial mechanism of phenolic compounds has not yet been elucidated, however, it is known that they have many action sites at the cellular level. Indeed, the presence of OH functional groups is relevant to the antibacterial activity of many phenolic compounds, which interact with bacteria’s cellular membranes by hydrogen bonding [62]. Thus, the membrane’s functions of nutrient uptake and enzyme activity would be affected, causing microbial cell death [22].

PPE-PLE obtained under optimal conditions shows a diameter of inhibition of 14 mm on *S. aureus*. This value was higher than the inhibition diameters obtained in the experimental design (i.e., 2 to 8.3 mm, Table 1). This result could be associated to the higher TPC of PPE-PLE obtained under optimal conditions. Although AMA of pomegranate peel extract has been mainly associated with ellagitannins such as punicalagin and ellagic acid [63], other phenolic compounds of PPE such as phenolic acids, flavonoids, proanthocyanidins, hydrolysable tannins, among others, could also exhibit antimicrobial synergistic effects towards different microorganisms such as bacteria, yeast, and molds [64]. Of note, it has been reported that a pomegranate peel extract containing a mixture of polyphenolic compounds showed better AMA than individually isolated compounds [14]. The use of PPE-PLE therefore showed promising results in AMA against *S. aureus*. Further research should be performed to evaluate the efficacy of PPE-PLE on the control of *S. aureus* in food applications.

#### 3.3.5. Cytotoxicity

**Figure 3:**
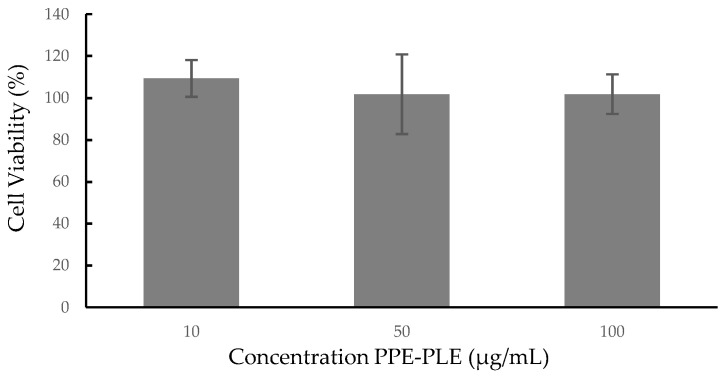
Cytotoxicity of PPE-PLE on human colorectal cancer cell line Caco-2.

Cellular cytotoxicity studies are an initial and essential step in determining the potential toxicity of a substance, including plant extracts or biologically active compounds isolated from them. In this study, the cytotoxic effects of PPE-PLE were investigated on the human colorectal cancer cell line Caco-2. As shown in Figure 3, the extract proved to be nontoxic to the cells at the three concentrations studied (10, 50 and 100 µg/mL), retaining 100% viability. A lower cell viability (50%) was reported on normal colon cells (CCD112) treated with 250 µg/mL of aqueous pomegranate peel extract [65]. The effects of hydroalcoholic pomegranate peel extract on different human cancer cell lines (HTB140, HTB177, MCF7, HCT116) and on MRC-5 normal fibroblasts, showed that the extract expressed selective cytotoxicity for cancer cells compared to a normal cell line [66]. According to these results, the concentration of extract and the type of cell line are factors that influence cell viability, therefore, it is necessary to carry out new studies using higher concentrations of PPE-PLE and other cell lines.

## 4. Conclusions

This research provides new insights into the efficacy of PLE on the recovery of bioactive compounds from pomegranate peel as material recycling. Of note, pomegranate peel represents 10.5% of the pomegranate fruit fresh weight. A combination of pressurized water and ethanol results in a PPE-PLE with a TPC and punicalagin content of 164.3 ± 10.7 mg GAE/g DW and 17 ± 3.6 mg/g DW obtained under optimal conditions. The combination of pressurized water and ethanol was not efficient for punicalagin recovery. Nevertheless, AMA and cytotoxicity findings showed promising results of PPE-PLE. Indeed, either polyphenol composition and other extracted bioactive compounds influenced the AMA. Further research in synergy or additive effects should be evaluated. The efficacy of PPE-PLE in food applications must continue to be studied in order to achieve adequate information on its potential for developing new food additives.

## Figures and Tables

**Table 1:** Central composite design and results of dependent variables in PLE from pomegranate peel.

Run	X_1_	X_2_	Temperature [X_1_]	Ethanol [X_2_]	TPC	Punicalagin	AMA
			(°C)	(%)	(mg GAE/g DW)	(mg/g DW)	*S. aureus* (mm)
1	−1	−1	55	10	14.1 ± 0.7	7.7 ± 0.2	2.0 ± 0.4
2	−1	1	55	90	75.9 ± 3.0	38.0 ± 1.9	7.5 ± 0.7
3	1	−1	185	10	73.0 ± 1.0	5.0 ± 0.2	4.0 ± 0.7
4	1	1	185	90	129.6 ± 6.3	30.1 ± 0.9	7.0 ± 2.8
5	−1.21	0	41.3	50	39.6 ± 1.6	36.1 ± 0.6	6.0 ± 0.0
6	+1.21	0	198.6	50	149.0 ± 5.3	22.0 ± 0.3	8.3 ± 1.1
7	0	−1.21	120	1.6	20.0 ± 3.5	4.0 ± 0.3	3.0 ± 0.7
8	0	+1.21	120	98.4	40.9 ± 1.9	23.3 ± 1.3	5.5 ± 1.4
9	0	0	120	50	39.2 ± 0.2	38.6 ± 2.5	4.8 ± 1.1
10	0	0	120	50	27.7 ± 0.5	35.0 ± 1.5	3.0 ± 0.7
11	0	0	120	50	33.2 ± 1.0	31.4 ± 0.6	3.8 ± 0.4
12	0	0	120	50	39.0 ± 0.2	38.0 ± 1.1	4.8 ± 0.4

Data represent mean ± standard deviation. TPC, total phenolic content; AMA, antimicrobial activity.

**Table 2:** Analysis of variance (ANOVA) for the pomegranate peel extract obtained by PLE.

Source	Sum of Squares	df	Mean Square	F-Ratio	*p*-Value	*R* ^2^	*R*^2^ df.adj
TPC							
X_1_: Temperature	8662.03	1	8662.03	288.84	0.0004 *	93.3	90.7
X_2_: Ethanol	2980.07	1	2980.07	99.37	0.0021 *		
X_1_X_1_	7179.23	1	7179.23	239.39	0.0006 *		
Lack of fit	1273.17	5	254.635	8.49	0.0542		
Pure error	899.675	3	299.892				
Total (corr.)	20184.5	11					
Punicalagin							
X_1_: Temperature	110.437	1	110.437	10.14	0.0499 *	91.1	87.7
X_2_: Ethanol	895.186	1	895.186	82.20	0.0028 *		
X_2_X_2_	777.825	1	777.825	71.43	0.0035 *		
Lack of fit	142.147	5	284.294	2.61	0.2297		
Pure error	32.67	3	10.89				
Total (corr.)	1958.27	11					
AMA							
X_1_: Temperature	257.347	1	257.347	3.60	0.1539	79.6	80.1
X_2_: Ethanol	191.717	1	191.717	26.84	0.0140 *		
X_1_X_1_	113.715	1	113.715	15.92	0.0282 *		
Lack of fit	635.145	5	127.029	1.78	0.3370		
Pure error	21.425	3	0.714167				
Total (corr.)	416.106	11					
Multiple Response Optimization							
Desirability	0.79896						
Factor	Low	High	Optimum				
X_1_: Temperature	41.3	198.6	198.6				
X_2_: Ethanol	1.6	98.4	76.6				

TPC, total phenolic content; AMA, antimicrobial activity; df, degrees of freedom; df.adj, adjusted degrees of freedom. * *p* < 0.05.

**Table 3:** Tentative identification of polar compounds and their derivatives in PPE-PLE by HPLC-DAD-ESI-TOF/MS.

**Peak**	**RT (min)**	***m*/*z* exp**	***m*/*z* cal**	**Molecular Formula**	**Tentative Compound**
1	4.2	181.0724	181.0718	C_6_H_13_O_6_	Sugar
2	4.8	353.0721	353.0725	C_12_H_17_O_12_	Sugar
3	5.9	353.0724	353.0878	C_16_H_17_O_9_	Chlorogenic acid
4	7.5	191.0195	191.0197	C_6_H_7_O_7_	Citric acid
5	8.0	191.0204	191.0197	C_6_H_7_O_7_	Isocitric acid
6	10.1	331.0657	331.0671	C_13_H_15_O_10_	Galloyl hexose isomer 1
7	10.9	125.0247	125.0244	C_6_H_5_O_3_	Phloroglucinol
8	11.3	331.0655	331.0.671	C_13_H_15_O_10_	Galloyl hexose isomer 2
9	11.7	541.0282	541.0260	C_24_H_13_O_15_	Punicalagin α
10	12.0	169.0143	169.0142	C_7_H_5_O_5_	Gallic acid
11	12.8	289.0869	289.0929	C_12_H_17_O_8_	Sugar derivative
12	13.3	541.0284	541.0260	C_24_H_13_O_15_	Punicalagin β
13	14.2	289.0927	289.0929	C_12_H_17_O_8_	Sugar derivative
14	15.2	541.0295	541.0260	C_24_H_13_O_15_	Punicalagin γ
15	16.5	469.0029	469.0049	C_21_H_9_O_13_	Valonic acid bilactone
16	17.3	461.0916	461.0937	C_18_H_21_O_14_	Unknown 1
17	17.7	219.0514	219.0510	C_8_H_11_O_7_	Citric acid, dimethyl ester
18	17.7	633.0726	633.0733	C_27_H_21_O_18_	Galloyl-HHDP-hexose
19	18.6	289.0718	289.0718	C_15_H_13_O_6_	Catechin
20	18.7	483.0768	483.0780	C_20_H_19_O_14_	Digalloyl hexoside
21	19.3	443.1938	443.1711	C_24_H_27_O_8_	Unknown 2
22	19.5	453.0963	453.0827	C_23_H_17_O_10_	Unknown 3
23	19.8	575.0125	575.0103	C_27_H_11_O_15_	Unknown 4
24	20.1	325.0915	325.0929	C_15_H_17_O_8_	*p*-Coumaric acid hexoside
25	21.3	633.0740	633.0463	C_27_H_21_O_18_	Corilagin
26	21.8	291.0168	291.0146	C_13_H_7_O_8_	Brevifolin carboxylic acid
27	22.6	431.1941	431.1711	C_23_H_27_O_8_	Unknown 5
28	24.1	345.0826	345.0827	C_14_H_17_O_10_	Methyl gallate hexoside
29	24.1	600.9896	600.9896	C_28_H_9_O_16_	Gallagyldilactone
30	24.4	197.0462	197.0455	C_9_H_10_O_5_	Gallic acid dimethyl ether
31	24.8	463.0526	463.0518	C_20_H_15_O_13_	Ellagic acid hexoside
32	24.9	275.0208	275.0197	C_13_H_7_O_7_	Decarboxyellagic acid
33	25.5	247.0258	247.0096	C_12_H_7_O_6_	Brevifolin
34	25.7	517.1526	517.1563	C_22_H_29_O_14_	Feruloyl sucrose
35	26.4	293.1034	293.1031	C_15_H_17_O_6_	Cinnamoyl rhamnoside
36	27.2	319.0446	319.0459	C_15_H_11_O_8_	Brevifolincarboxylic acid ethyl ester
37	27.6	491.0845	491.0620	C_25_H_15_O_11_	Unknown 6
38	27.9	425.0161	425.0150	C_20_H_9_O_11_	Monodecarboxyvaloneic acid dilactone
39	28.1	463.0906	463.0882	C_21_H_19_O_12_	Hyperoside
**Peak**	**RT (min)**	***m*/*z* exp**	***m*/*z* cal**	**Molecular Formula**	**Tentative Identification**
40	28.3	433.0418	433.0412	C_19_H_13_O_12_	Ellagic acid pentoside
41	29.0	447.0594	447.0569	C_20_H_15_O_12_	Ellagic acid-deoxyhexoside
42	29.0	181.0505	181.0506	C_9_H_9_O_4_	Homovanillic acid
43	30.0	300.9998	300.9990	C_14_H_5_O_8_	Ellagic acid
44	31.9	447.0908	447.0933	C_21_H_19_O_11_	Luteolin hexoside
45	32.4	321.1344	321.1344	C_17_H_21_O_6_	Unknown 7
46	33.9	417.0822	417.0827	C_20_H_17_O_10_	Luteolin pentoside
47	34.5	285.0431	285.0557	C_15_H_9_O_6_	Kaempferol
48	35.1	329.0305	329.0303	C_16_H_9_O_8_	Ellagic acid dimethyl ether
49	36.2	285.0419	285.0405	C_15_H_9_O_6_	Luteolin
50	36.7	317.0406	371.0409	C_18_H_11_O_9_	Unknown 8
51	38.0	463.0654	463.0671	C_24_H_15_O_10_	Unknown 9

## Data Availability

The data presented in this study are available on request from the corresponding author. The data are not publicly available due to privacy restrictions.
